# *catena*-Poly[bis­[(1,10-phenanthroline)cobalt(II)]-μ_4_-3,6-dicarb­oxy­cyclo­hexane-1,2,4,5-tetra­carboxyl­ato]

**DOI:** 10.1107/S1600536811019970

**Published:** 2011-06-04

**Authors:** Wei Xu

**Affiliations:** a Center of Applied Solid State Chemistry Research, Ningbo University, Ningbo 315211, People’s Republic of China

## Abstract

In the title compound, [Co_2_(C_12_H_8_O_12_)(C_12_H_8_N_2_)_2_]_*n*_, each 3,6-dicarb­oxy­cyclo­hexane-1,2,4,5-tetra­carboxyl­ate (H_2_chhc^4−^) anion has crystallographically imposed *C*_2_ symmetry and bridges four six-coordinate Co atoms, generating polymeric chains running along [010]. These chains are further extended into a three-dimensional network *via* O—H⋯O hydrogen-bonding inter­actions and inter­chain π–π stacking inter­actions [centroid–centroid distance = 3.662 (2) Å].

## Related literature

For the design and synthesis of coordination polymer complexes and their potential applications, see: Biradha *et al.* (2006 ▶); Bauer *et al.* (2007 ▶); Zacher *et al.* (2011 ▶). For the 1,2,3,4,5,6-cyclo­hexa­nehexa­carboxyl­ate ligand, see: Li *et al.* (2006 ▶); Wang *et al.* (2008 ▶); Thuéry & Masci (2010 ▶). For related structures, see: Konar *et al.* (2004 ▶); Li *et al.* (2006 ▶). 
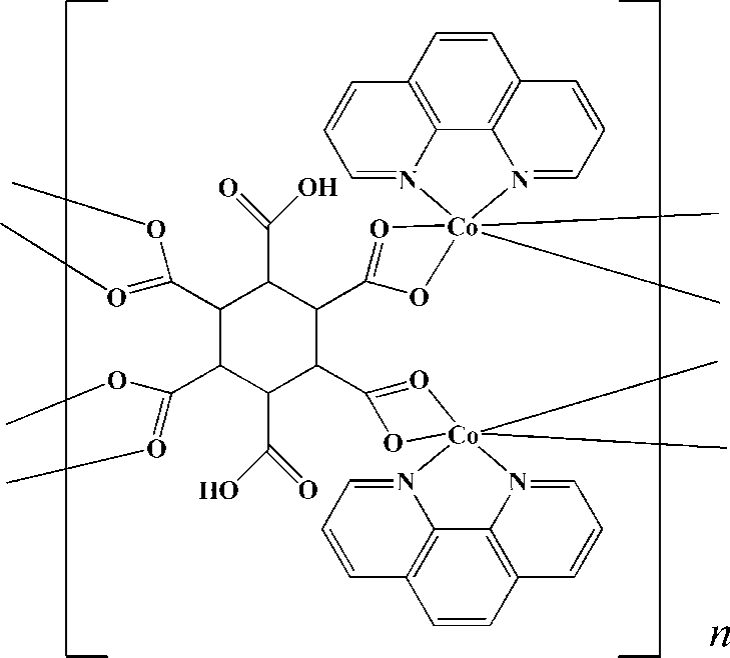


## Experimental

### 

#### Crystal data

[Co_2_(C_12_H_8_O_12_)(C_12_H_8_N_2_)_2_]
*M* *_r_* = 822.46
Monoclinic, 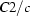

*a* = 22.180 (4) Å
*b* = 8.9520 (18) Å
*c* = 16.426 (3) Å
β = 93.33 (3)°
*V* = 3256.0 (11) Å^3^
*Z* = 4
Mo *K*α radiation
μ = 1.10 mm^−1^
*T* = 295 K
0.31 × 0.23 × 0.15 mm

#### Data collection

Siemens P4 diffractometer
Absorption correction: ψ scan (*XSCANS*; Siemens, 1996 ▶) *T*_min_ = 0.702, *T*_max_ = 0.784
4566 measured reflections
3753 independent reflections
3312 reflections with *I* > 2σ(*I*)
*R*_int_ = 0.022
3 standard reflections every 97 reflections  intensity decay: none

#### Refinement

*R*[*F* ^2^ > 2σ(*F* ^2^)] = 0.031
*wR*(*F* ^2^) = 0.083
*S* = 1.03
3753 reflections
248 parameters
H atoms treated by a mixture of independent and constrained refinement
Δρ_max_ = 0.38 e Å^−3^
Δρ_min_ = −0.33 e Å^−3^

### 

Data collection: *XSCANS* (Siemens, 1996 ▶); cell refinement: *XSCANS*; data reduction: *XSCANS*; program(s) used to solve structure: *SHELXS97* (Sheldrick, 2008 ▶); program(s) used to refine structure: *SHELXL97* (Sheldrick, 2008 ▶); molecular graphics: *SHELXTL* (Sheldrick, 2008 ▶); software used to prepare material for publication: *SHELXL97*.

## Supplementary Material

Crystal structure: contains datablock(s) global, I. DOI: 10.1107/S1600536811019970/sj5153sup1.cif

Structure factors: contains datablock(s) I. DOI: 10.1107/S1600536811019970/sj5153Isup2.hkl

Additional supplementary materials:  crystallographic information; 3D view; checkCIF report

## Figures and Tables

**Table 1:** Selected bond lengths (Å)

Co1—O1	2.2002 (13)
Co1—O2	2.0890 (13)
Co1—O5^i^	2.1211 (13)
Co1—O6^i^	2.1519 (13)
Co1—N1	2.1012 (15)
Co1—N2	2.1016 (15)

Symmetry code: (i) 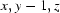.

**Table 2:** Hydrogen-bond geometry (Å, °)

*D*—H⋯*A*	*D*—H	H⋯*A*	*D*⋯*A*	*D*—H⋯*A*
O4—H4*A*⋯O2^ii^	0.79 (3)	1.89 (3)	2.627 (2)	156 (2)

Symmetry code: (ii) 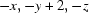.

**Fig. 1.**
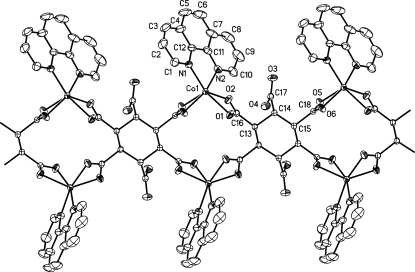
ORTEP view of the polymer chain [Co2(phen)2(H2chhc)]n of the title complex. The displacement ellipsoids are drawn at 40% probability level, hydrogen atoms are omitted for clarity.

**Fig. 2.**
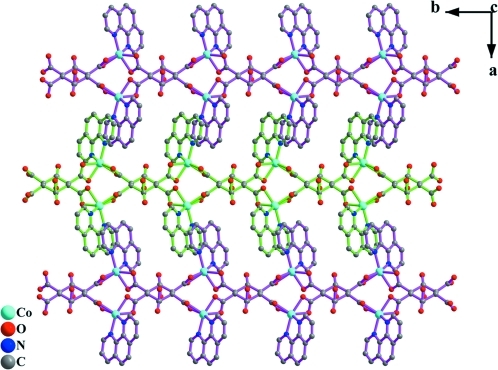
A view of a single layer of the title complex.

## supplementary crystallographic information

### Comment

The rational design and construction of metal-organic coordination polymers with flexible multidentate ligands have received more and more attention due to their intriguing structural topologies and novel properties for potential applications (Biradha, *et al.*, 2006; Bauer, *et al.*, 2007; Zacher, *et al.*, 2011). As a typical flexible cycloalkane polycarboxylic acid ligand, we have focused on the 1,2,3,4,5,6-cyclohexanehexacarboxylic acid (H_6_chhc) whose coordination chemistry remains practically unexplored. We were particularly aware that the greater flexibility of this ligand would make the prediction and control of the final coordination networks that it generates more difficult.  (Wang, *et al.*, 2008; Thuéry & Masci, 2010). Herein, we report a new cobalt coordination polymer, [Co_2_(phen)_2_(H_2_chhc)]_n_, resulting from reaction of Co^2+^ cations, phen and H_6_chhc under hydrothermal conditions. It is isostructural with the previously reported [Ni_2_(phen)_2_(H_2_chhc)]_n_ complex (Li, *et al.*, 2006).

The asymmetric unit of the title compound consists of one Co^2+^ cation, one phen ligand and one-half of a H_2_chhc^4-^ anion lying across a twofold rotation axis. The Co atoms are each in an octahedral environment defined by two N atoms of one phen ligand and four O atoms of two carboxylate groups from different H_2_chhc^4-^ anions. The Co-O bond lengths fall in the range 2.089 (1)-2.200 (1) Å and the two Co-N distances are 2.101 (2) and 2.102 (2) Å (Table 1), thus falling in the expected region (Konar, *et al.*, 2004). The octahedral coordination around the Co atoms are strongly distorted since the diametrical and non-diametrical bond angles indicate signficant deviations from 180° and 90°, respectively. The H_2_chhc^4-^ ligands assume an *e,e,e,e,e,e*-conformation with the central ring adopting a chair-shaped configuration, the carboxylate and carboxyl groups being located at the equatorical sites. Each carboxylate group of the H_2_chhc^4-^ anion chelates one Co atom. As a result, the H_2_chhc^4-^ anions are each coordinated to four [Co(phen)]^2+^ units, leading to polymeric chains [Co_2_(phen)_2_(H_2_chhc)]_n_ running along the [010] direction with the phen ligands *exo*-orientated (Fig. 1). The phen ligands of two adjacent supramolecular chains are stacked *via* the quinoline fragments (centroid-centroid distance = 3.662 (2) Å). Obviously, such π-π stacking interactions are responsible for the supramolecular assembly of the one-dimensional chains into two-dimensional layers parallel to (001) (Fig. 2). The layers are further connected to form a three-dimensional framework *via* interlayer O-H···O hydrogen bonds (d(O4···O2^#1^ = 2.627 (2) Å, <O4-H4A···O2^#1^ = 156 (2)°, #1 = -x, 2-y, -z).

### Experimental

CoCl_2_.6H_2_O (0.238 g, 1.0 mmol), H_6_chhc (0.173 g, 0.5 mmol), phen (0.200 g, 1.0 mmol) and NaOH 1.5 mL (1 M) were stirred in 20 mL H_2_O. The resulting mixture was placed in a 23 mL Teflon-lined autoclave and heated at 170 °C for 3 days. The reaction system was cooled to room temperature at a rate of 20 °C/h, and small amount of pink crystals of the title complex was obtained.

### Refinement

All H atoms bound to C were position geometrically and refined as riding, with C-H = 0.93 Å and *U*_iso_(H) = 1.2*U*_eq_(C). H atoms attached to O were located in difference Fourier maps and refined freely with *U*_iso_(H) = 1.5*U*_eq_(O).

### Crystal data

**Table:**

[Co_2_(C_12_H_8_O_12_)(C_12_H_8_N_2_)_2_]	*F*(000) = 1672
*M**_r_* = 822.46	*D*_x_ = 1.678 Mg m^−^^3^
Monoclinic, *C*2/*c*	Mo *K*α radiation, λ = 0.71073 Å
Hall symbol: -C 2yc	Cell parameters from 25 reflections
*a* = 22.180 (4) Å	θ = 5.0–12.5°
*b* = 8.9520 (18) Å	µ = 1.10 mm^−^^1^
*c* = 16.426 (3) Å	*T* = 295 K
β = 93.33 (3)°	Block, pink
*V* = 3256.0 (11) Å^3^	0.31 × 0.23 × 0.15 mm
*Z* = 4	

### Data collection

**Table:**

Siemens P4 diffractometer	3312 reflections with *I* > 2σ(*I*)
Radiation source: fine-focus sealed tube	*R*_int_ = 0.022
graphite	θ_max_ = 27.5°, θ_min_ = 2.5°
θ/2θ scans	*h* = −28→1
Absorption correction: ψ scan (*XSCANS*; Siemens, 1996)	*k* = −1→11
*T*_min_ = 0.702, *T*_max_ = 0.784	*l* = −21→21
4566 measured reflections	3 standard reflections every 97 reflections
3753 independent reflections	intensity decay: none

### Refinement

**Table:**

Refinement on *F*^2^	Secondary atom site location: difference Fourier map
Least-squares matrix: full	Hydrogen site location: inferred from neighbouring sites
*R*[*F*^2^ > 2σ(*F*^2^)] = 0.031	H atoms treated by a mixture of independent and constrained refinement
*wR*(*F*^2^) = 0.083	*w* = 1/[σ^2^(*F*_o_^2^) + (0.0413*P*)^2^ + 2.2781*P*] where *P* = (*F*_o_^2^ + 2*F*_c_^2^)/3
*S* = 1.03	(Δ/σ)_max_ < 0.001
3753 reflections	Δρ_max_ = 0.38 e Å^−^^3^
248 parameters	Δρ_min_ = −0.33 e Å^−^^3^
0 restraints	Extinction correction: *SHELXL97* (Sheldrick, 2008), Fc^*^=kFc[1+0.001xFc^2^λ^3^/sin(2θ)]^-1/4^
Primary atom site location: structure-invariant direct methods	Extinction coefficient: 0.00077 (19)

### Special details

**Table:**

Geometry. All esds (except the esd in the dihedral angle between two l.s. planes) are estimated using the full covariance matrix. The cell esds are taken into account individually in the estimation of esds in distances, angles and torsion angles; correlations between esds in cell parameters are only used when they are defined by crystal symmetry. An approximate (isotropic) treatment of cell esds is used for estimating esds involving l.s. planes.
Refinement. Refinement of F^2^ against ALL reflections. The weighted R-factor wR and goodness of fit S are based on F^2^, conventional R-factors R are based on F, with F set to zero for negative F^2^. The threshold expression of F^2^ > 2sigma(F^2^) is used only for calculating R-factors(gt) etc. and is not relevant to the choice of reflections for refinement. R-factors based on F^2^ are statistically about twice as large as those based on F, and R- factors based on ALL data will be even larger.

### Fractional atomic coordinates and isotropic or equivalent isotropic displacement parameters (Å^2^)

**Table:**

	*x*	*y*	*z*	*U*_iso_*/*U*_eq_	
Co1	0.098444 (9)	0.64743 (2)	0.140367 (13)	0.02138 (9)	
O1	0.07208 (6)	0.79951 (15)	0.23735 (7)	0.0330 (3)	
O2	0.04781 (7)	0.83776 (14)	0.10892 (7)	0.0327 (3)	
O3	0.08305 (7)	1.16900 (18)	0.04746 (9)	0.0433 (4)	
O4	−0.01738 (6)	1.14637 (17)	0.04776 (8)	0.0363 (3)	
H4A	−0.0159 (12)	1.148 (3)	−0.0004 (19)	0.054*	
O5	0.09100 (5)	1.45307 (15)	0.21296 (8)	0.0314 (3)	
O6	0.01745 (5)	1.51543 (15)	0.12579 (8)	0.0329 (3)	
N1	0.13438 (6)	0.55177 (17)	0.03690 (9)	0.0277 (3)	
N2	0.18993 (7)	0.70526 (19)	0.16045 (9)	0.0322 (3)	
C1	0.10617 (9)	0.4793 (2)	−0.02411 (11)	0.0378 (4)	
H1A	0.0642	0.4771	−0.0272	0.045*	
C2	0.13708 (13)	0.4053 (3)	−0.08457 (14)	0.0544 (6)	
H2A	0.1159	0.3563	−0.1272	0.065*	
C3	0.19861 (13)	0.4065 (3)	−0.07970 (15)	0.0591 (7)	
H3A	0.2196	0.3561	−0.1186	0.071*	
C4	0.23052 (10)	0.4833 (3)	−0.01624 (14)	0.0472 (5)	
C5	0.29531 (12)	0.4955 (4)	−0.00706 (19)	0.0673 (8)	
H5A	0.3186	0.4468	−0.0441	0.081*	
C6	0.32300 (10)	0.5748 (4)	0.05321 (19)	0.0686 (9)	
H6A	0.3649	0.5806	0.0569	0.082*	
C7	0.28897 (9)	0.6512 (3)	0.11216 (16)	0.0517 (6)	
C8	0.31442 (11)	0.7393 (4)	0.17623 (18)	0.0660 (8)	
H8A	0.3561	0.7516	0.1821	0.079*	
C9	0.27855 (12)	0.8067 (4)	0.22961 (17)	0.0658 (8)	
H9A	0.2954	0.8650	0.2719	0.079*	
C10	0.21599 (11)	0.7875 (3)	0.22011 (14)	0.0487 (5)	
H10A	0.1917	0.8338	0.2568	0.058*	
C11	0.22540 (8)	0.6391 (2)	0.10648 (12)	0.0339 (4)	
C12	0.19595 (8)	0.5557 (2)	0.04106 (11)	0.0318 (4)	
C13	0.00497 (7)	1.00717 (17)	0.20407 (9)	0.0213 (3)	
H13A	−0.0344	0.9985	0.1741	0.026*	
C14	0.03623 (7)	1.15052 (16)	0.17600 (9)	0.0205 (3)	
H14A	0.0778	1.1519	0.1998	0.025*	
C15	0.00349 (7)	1.29201 (17)	0.20377 (9)	0.0194 (3)	
H15A	−0.0367	1.2958	0.1758	0.023*	
C16	0.04335 (8)	0.87293 (17)	0.18323 (10)	0.0234 (3)	
C17	0.03790 (8)	1.15477 (18)	0.08330 (10)	0.0256 (3)	
C18	0.03920 (7)	1.42928 (17)	0.17948 (9)	0.0209 (3)	

### Atomic displacement parameters (Å^2^)

**Table:**

	*U*^11^	*U*^22^	*U*^33^	*U*^12^	*U*^13^	*U*^23^
Co1	0.02179 (13)	0.01718 (13)	0.02545 (13)	−0.00066 (8)	0.00385 (8)	−0.00008 (8)
O1	0.0451 (7)	0.0284 (6)	0.0258 (6)	0.0125 (6)	0.0031 (5)	0.0002 (5)
O2	0.0497 (8)	0.0259 (6)	0.0228 (6)	0.0115 (6)	0.0048 (5)	−0.0013 (5)
O3	0.0426 (8)	0.0505 (9)	0.0389 (7)	0.0011 (7)	0.0212 (6)	0.0015 (6)
O4	0.0402 (7)	0.0477 (9)	0.0209 (6)	0.0031 (6)	0.0014 (5)	−0.0005 (6)
O5	0.0286 (6)	0.0283 (6)	0.0368 (6)	−0.0066 (5)	−0.0040 (5)	0.0084 (5)
O6	0.0274 (6)	0.0276 (6)	0.0431 (7)	−0.0037 (5)	−0.0031 (5)	0.0141 (6)
N1	0.0265 (7)	0.0298 (7)	0.0272 (7)	0.0006 (6)	0.0044 (5)	−0.0008 (6)
N2	0.0299 (7)	0.0335 (8)	0.0328 (7)	−0.0094 (6)	−0.0004 (6)	0.0029 (7)
C1	0.0407 (10)	0.0412 (11)	0.0313 (9)	−0.0015 (8)	0.0005 (7)	−0.0037 (8)
C2	0.0764 (17)	0.0522 (14)	0.0353 (11)	−0.0025 (13)	0.0089 (10)	−0.0145 (10)
C3	0.0742 (17)	0.0589 (15)	0.0469 (13)	0.0132 (14)	0.0256 (12)	−0.0108 (12)
C4	0.0444 (11)	0.0513 (13)	0.0479 (12)	0.0140 (10)	0.0206 (9)	0.0045 (10)
C5	0.0411 (13)	0.086 (2)	0.0782 (18)	0.0230 (14)	0.0307 (13)	0.0099 (17)
C6	0.0245 (10)	0.096 (2)	0.087 (2)	0.0121 (13)	0.0166 (11)	0.0225 (18)
C7	0.0236 (9)	0.0692 (17)	0.0620 (14)	−0.0055 (9)	0.0005 (9)	0.0216 (12)
C8	0.0310 (11)	0.092 (2)	0.0729 (17)	−0.0238 (13)	−0.0132 (11)	0.0211 (16)
C9	0.0571 (15)	0.081 (2)	0.0564 (15)	−0.0368 (15)	−0.0180 (12)	0.0040 (14)
C10	0.0505 (13)	0.0511 (13)	0.0438 (11)	−0.0208 (11)	−0.0043 (9)	−0.0023 (10)
C11	0.0229 (8)	0.0386 (10)	0.0404 (10)	−0.0019 (7)	0.0025 (7)	0.0106 (8)
C12	0.0278 (8)	0.0337 (9)	0.0348 (9)	0.0038 (7)	0.0091 (7)	0.0062 (8)
C13	0.0275 (7)	0.0155 (7)	0.0213 (7)	−0.0008 (6)	0.0037 (6)	−0.0001 (6)
C14	0.0228 (7)	0.0161 (7)	0.0227 (7)	−0.0001 (6)	0.0036 (5)	0.0001 (6)
C15	0.0209 (7)	0.0157 (7)	0.0217 (7)	0.0000 (5)	0.0020 (5)	0.0003 (6)
C16	0.0302 (8)	0.0168 (7)	0.0238 (7)	−0.0003 (6)	0.0051 (6)	0.0008 (6)
C17	0.0342 (8)	0.0179 (7)	0.0252 (8)	0.0015 (6)	0.0077 (6)	0.0009 (6)
C18	0.0238 (7)	0.0167 (7)	0.0228 (7)	0.0008 (6)	0.0058 (6)	−0.0014 (6)

### Geometric parameters (Å, °)

**Table:**

Co1—O1	2.2002 (13)	C3—H3A	0.9300
Co1—O2	2.0890 (13)	C4—C12	1.406 (3)
Co1—O5^i^	2.1211 (13)	C4—C5	1.440 (3)
Co1—O6^i^	2.1519 (13)	C5—C6	1.338 (5)
Co1—N1	2.1012 (15)	C5—H5A	0.9300
Co1—N2	2.1016 (15)	C6—C7	1.435 (4)
Co1—C18^i^	2.4599 (16)	C6—H6A	0.9300
Co1—C16	2.4827 (16)	C7—C8	1.407 (4)
O1—C16	1.250 (2)	C7—C11	1.412 (3)
O2—C16	1.270 (2)	C8—C9	1.359 (4)
O3—C17	1.198 (2)	C8—H8A	0.9300
O4—C17	1.329 (2)	C9—C10	1.398 (3)
O4—H4A	0.79 (3)	C9—H9A	0.9300
O5—C18	1.263 (2)	C10—H10A	0.9300
O5—Co1^ii^	2.1211 (13)	C11—C12	1.435 (3)
O6—C18	1.247 (2)	C13—C16	1.523 (2)
O6—Co1^ii^	2.1519 (13)	C13—C13^iii^	1.538 (3)
N1—C1	1.321 (2)	C13—C14	1.542 (2)
N1—C12	1.364 (2)	C13—H13A	0.9800
N2—C10	1.330 (3)	C14—C17	1.526 (2)
N2—C11	1.355 (3)	C14—C15	1.542 (2)
C1—C2	1.405 (3)	C14—H14A	0.9800
C1—H1A	0.9300	C15—C18	1.528 (2)
C2—C3	1.363 (4)	C15—C15^iii^	1.535 (3)
C2—H2A	0.9300	C15—H15A	0.9800
C3—C4	1.405 (4)	C18—Co1^ii^	2.4599 (16)
			
O2—Co1—N1	110.87 (6)	C5—C6—C7	121.0 (2)
O2—Co1—N2	109.77 (6)	C5—C6—H6A	119.5
N1—Co1—N2	79.58 (6)	C7—C6—H6A	119.5
O2—Co1—O5^i^	138.57 (6)	C8—C7—C11	116.6 (2)
N1—Co1—O5^i^	99.55 (6)	C8—C7—C6	124.6 (2)
N2—Co1—O5^i^	102.70 (6)	C11—C7—C6	118.8 (2)
O2—Co1—O6^i^	89.26 (5)	C9—C8—C7	120.5 (2)
N1—Co1—O6^i^	92.27 (6)	C9—C8—H8A	119.8
N2—Co1—O6^i^	160.89 (6)	C7—C8—H8A	119.8
O5^i^—Co1—O6^i^	61.34 (5)	C8—C9—C10	119.2 (2)
O2—Co1—O1	60.84 (5)	C8—C9—H9A	120.4
N1—Co1—O1	165.26 (6)	C10—C9—H9A	120.4
N2—Co1—O1	91.64 (6)	N2—C10—C9	122.4 (2)
O5^i^—Co1—O1	93.89 (5)	N2—C10—H10A	118.8
O6^i^—Co1—O1	99.50 (6)	C9—C10—H10A	118.8
O2—Co1—C18^i^	115.12 (6)	N2—C11—C7	122.6 (2)
N1—Co1—C18^i^	96.96 (6)	N2—C11—C12	117.47 (15)
N2—Co1—C18^i^	132.85 (6)	C7—C11—C12	119.9 (2)
O5^i^—Co1—C18^i^	30.88 (5)	N1—C12—C4	122.56 (19)
O6^i^—Co1—C18^i^	30.46 (5)	N1—C12—C11	117.50 (16)
O1—Co1—C18^i^	97.68 (5)	C4—C12—C11	119.94 (18)
O2—Co1—C16	30.74 (5)	C16—C13—C13^iii^	109.53 (11)
N1—Co1—C16	140.98 (6)	C16—C13—C14	108.82 (12)
N2—Co1—C16	104.00 (6)	C13^iii^—C13—C14	112.70 (10)
O5^i^—Co1—C16	116.86 (6)	C16—C13—H13A	108.6
O6^i^—Co1—C16	93.24 (6)	C13^iii^—C13—H13A	108.6
O1—Co1—C16	30.20 (5)	C14—C13—H13A	108.6
C18^i^—Co1—C16	106.99 (5)	C17—C14—C13	110.89 (13)
C16—O1—Co1	87.50 (10)	C17—C14—C15	108.29 (12)
C16—O2—Co1	92.02 (10)	C13—C14—C15	111.56 (12)
C17—O4—H4A	110 (2)	C17—C14—H14A	108.7
C18—O5—Co1^ii^	89.55 (10)	C13—C14—H14A	108.7
C18—O6—Co1^ii^	88.55 (10)	C15—C14—H14A	108.7
C1—N1—C12	118.65 (16)	C18—C15—C15^iii^	109.99 (10)
C1—N1—Co1	128.96 (13)	C18—C15—C14	108.86 (12)
C12—N1—Co1	112.02 (12)	C15^iii^—C15—C14	111.64 (10)
C10—N2—C11	118.66 (18)	C18—C15—H15A	108.8
C10—N2—Co1	128.76 (15)	C15^iii^—C15—H15A	108.8
C11—N2—Co1	112.44 (12)	C14—C15—H15A	108.8
N1—C1—C2	122.6 (2)	O1—C16—O2	119.22 (15)
N1—C1—H1A	118.7	O1—C16—C13	121.57 (14)
C2—C1—H1A	118.7	O2—C16—C13	119.17 (14)
C3—C2—C1	118.9 (2)	O1—C16—Co1	62.30 (9)
C3—C2—H2A	120.6	O2—C16—Co1	57.23 (8)
C1—C2—H2A	120.6	C13—C16—Co1	174.86 (12)
C2—C3—C4	120.5 (2)	O3—C17—O4	124.52 (17)
C2—C3—H3A	119.8	O3—C17—C14	124.24 (17)
C4—C3—H3A	119.8	O4—C17—C14	111.20 (14)
C3—C4—C12	116.8 (2)	O6—C18—O5	120.56 (15)
C3—C4—C5	124.8 (2)	O6—C18—C15	119.79 (14)
C12—C4—C5	118.3 (2)	O5—C18—C15	119.64 (14)
C6—C5—C4	122.0 (2)	O6—C18—Co1^ii^	60.99 (9)
C6—C5—H5A	119.0	O5—C18—Co1^ii^	59.57 (8)
C4—C5—H5A	119.0	C15—C18—Co1^ii^	178.92 (11)

Symmetry codes: (i) *x*, *y*−1, *z*; (ii) *x*, *y*+1, *z*; (iii) −*x*, *y*, −*z*+1/2.

### Hydrogen-bond geometry (Å, °)

**Table:**

*D*—H···*A*	*D*—H	H···*A*	*D*···*A*	*D*—H···*A*
O4—H4A···O2^iv^	0.79 (3)	1.89 (3)	2.627 (2)	156 (2)

Symmetry codes: (iv) −*x*, −*y*+2, −*z*.
